# An In Vitro Pilot Study on the Effects of Silver Diamine Fluoride on Periodontal Pathogens and Three-Dimensional Scaffolds of Human Fibroblasts and Epithelial Cells

**DOI:** 10.1155/2022/9439096

**Published:** 2022-05-17

**Authors:** Yolanda Ho, Robert Gyurko, Naciye Guzin Uzel, Bjorn Steffensen, Pinelopi Xenoudi, Cheen Loo, Driss Zoukhri

**Affiliations:** ^1^Department of Periodontology, Tufts University School of Dental Medicine, Boston, MA, USA; ^2^College of Dental Medicine, California Northstate University, Sacramento, CA, USA; ^3^Department of Pediatric Dentistry, Tufts University School of Dental Medicine, Boston, MA, USA; ^4^Department of Comprehensive Care, Tufts University School of Dental Medicine, Boston, MA, USA

## Abstract

**Objective:**

The aims of this study were to investigate the antibacterial and cytotoxic effects of silver diamine fluoride (SDF) on periodontal pathogens and human skin constructs, respectively.

**Background:**

SDF has been proven to have bactericidal effects on cariogenic bacteria. No studies to date evaluated the bactericidal effects of SDF on periodontal pathogens nor its effect on epithelium and fibroblasts.

**Methods:**

*Streptococcus mutans, Porphyromonas gingivalis,* and *Aggregatibacter actinomycetemcomitans* were cultured in monospecies biofilms, exposed to increasing concentrations of SDF and inoculated on agar plates to assess viability. Human gingival fibroblasts in 2D cultures were exposed to 1 *μ*L of 0.394% of SDF and viewed using real-time imaging. Finally, SDF was applied to human, 3D tissue scaffolds of fibroblasts and keratinocytes, and termed human skin equivalents (HSE). A clinical dose of 38% SDF was applied, and HSE were cultured for 12 hours, 1, 3, 5, and 10 days. The tissue was observed clinically and histologically with hematoxylin and eosin staining and TUNEL.

**Results:**

*S. mutans* and *A. actinomycetemcomitans* growth was completely inhibited using all dilutions of SDF, whereas *P. gingivalis* was still viable with 0.197% and 0.098% of SDF. Single-layer fibroblasts experienced immediate necrosis upon contact with SDF. Application of SDF to HSE showed maturation of a whitish lesion within 24 hours, followed by pigmented, crusted tissue after 3 days. Histological evaluation of treated tissues showed apoptotic cells in the epithelium and upper half of the connective tissue.

**Conclusion:**

Our data suggest that SDF has bactericidal properties against two periodontal pathogens: *P. gingivalis* and *A. actinomycetemcomitans*. SDF caused immediate necrosis of monolayer fibroblasts, but does not extend to the full extent of layered fibroblasts in HSE.

## 1. Introduction

The periodontium, anchoring the dentition within the alveolar process, is made up of the gingiva, cementum, periodontal ligament, and alveolar bone. Periodontal disease is an inflammatory process resulting from an imbalance between the host response and bacterial equilibrium. While there are many factors that contribute to the pathogenicity of periodontal disease, the main contributor is bacteria. Thus, the primary pathogenic etiology in periodontology mirrors cardiology in multispecies bacterial biofilms. First, cariogenic bacteria, like Gram-positive cocci, populate the supragingival plaque and next, Gram-negative cocci and rods, followed by filamentous forms [1]. The host response will react to these bacterial pathogens in the initial and early forms of gingivitis involving recruitment of neutrophils and lymphocytes, whereas an established lesion exhibits plasma cells [2]. However, the distinguishing feature of the advanced lesion (periodontitis) comes with loss of attachment. As the topography changes, there is a dysbiotic shift of the biofilm, resulting in growth of anaerobic, mobile, Gram-negative bacteria. This shift in periodontitis is marked by keystone pathogen, like *Porphyromonas gingivalis* [3]. *P. gingivalis* is capable of recruiting toll-like receptor-2 (TLR2). TLR2 identifies pathogen-associated molecular patterns (PAMPs) and signals the immune response of the adaptive immune system, along with components of the complement system with C5a [4]. By utilizing these mechanisms, *P. gingivalis* may act outside of prototypical proinflammatory roles, and a small number can disproportionately affect the host [3].

The traditional regimen for the treatment of periodontal disease involves elimination of biofilm through mechanical and therapeutic means (e.g., local or systemic antibiotics or antiseptics). The majority of patients respond well to conventional therapy, but a subset of patients (0.5–4%) experience persistent, destructive periodontal disease [5]. The term, “refractory periodontitis” (RP), coined in the 1989 World Workshop in Clinical Periodontics, was coined to describe these patients. The term overgeneralizes a multifactorial disease entity, but it is helpful in referencing these patients who demonstrate additional attachment loss even with strict maintenance [6]. Antibiotics are currently the adjunctive standard for the treatment of patients with RP [7]. However, there is no standard antibiotic regiment due to the heterogeneous microbiological profile [8]. The heterogeneity of RP poses a problem to antibiotic therapy—an improper pairing may lead to ineffective or potentially harmful effects.

The U.S. Food and Drug Administration (FDA) recently approved SDF (Ag (NH3)2F) for the treatment of dentinal sensitivity and interim caries arresting medicament. SDF is a colorless, odorless solution that plugs dentin tubules with a protective layer of a high concentration of aqueous silver [9]. SDF (38%) inhibits the development of a number of cariogenic bacteria including *Streptococcus mutans*, *Actinomyces naeslundii, Lactobacillus acidophilus,* and *Lacticaseibacillus rhamnosus* [10, 11].

Cytotoxic effects of SDF on human gingival fibroblasts have been demonstrated [12]. The primary SDF application protocol from the University of California has described a transient, white lesion on gingiva exposed to SDF [13]. Others have substantiated clinical observation of a mildly painful, white lesion forming and dissipating within 48 hours [14]. A researcher in Peru took intraoral photos (before application, immediately after, 24 hours after, and 1 week after). The gingiva was assigned an “erythema score” and categorized with Loe's gingival index. Reports showed an initial increase in inflammation within 24-hours, only to resolve soon after [15]. In the present studies, effects of SDF on fibroblasts and epithelial cells were studied using 2D and 3D culture systems. The aims of the present study were to investigate both the antibacterial and cytotoxic effects of silver diamine fluoride.

## 2. Materials and Methods

### 2.1. Bacterial Strain and Inoculum Preparation

Bacterial strains were purchased from American Type Culture Collection (ATCC) (Manassas, VA). Three bacterial strains were used: *S. mutans* (ATCC 25175), *Aggregatibacter actinomycetemcomitans* (ATCC 33384), and *P. gingivalis* (ATCC 33277). *S. mutans* and *A. actinomycetemcomitans* were grown on Bacto™ Brain Heart Infusion agar, and *P. gingivalis* on Bacto™ Tryptic soy agar with 5% defibrinated sheep blood. All bacteria were grown at 37°C with: *S. mutans* in aerobic conditions, *A. actinomycetemcomitans* in microaerophilic condition (5% CO_2_), and *P. gingivalis* in anaerobic conditions. Colonies were isolated using the quadrant streak plate method (in triplicates) and incubated: *S. mutans*—24 hours—and *A. actinomycetemcomitans* and *P. gingivalis*—72 hours. Single colonies were selected from each plate. The bacteria were cultured in liquid broth and serially diluted to an optical density of 600 nm, which resulted in a concentration of 10^6^ CFU/mL.

### 2.2. Silver Diamine Fluoride Preparation and Bacterial Culture

Advantage Arrest Silver Diamine fluoride 38% was obtained from Elevate Oral Care (West Palm Beach, FL). Starting from the initial 38% SDF solution, a two-fold serial dilution series, in sterile water, was used, resulting in eight concentrations to be tested: 12.6%, 6.3%, 3.15%, 1.57%, 0.788%, 0.394%, 0.197%, and 0.098%. Dilutions were immediately used following preparation, and the stock solution was kept in a sterile, opaque container at room temperature. A 96-well plate (Fisherbrand 96 well plates sterile, Fisher Scientific, Leicestershire, UK) was used for each bacterium (in duplicate). Each well received 10 *μ*L of bacterial inoculum, 140 *μ*L of broth, and 50 *μ*L of SDF with each respective dilution. For each bacterium, two positive controls were used with 50 *μ*L of CHX in place of SDF, and two negative controls were used with 50 *μ*L of additional broth in place of SDF. Plates were covered with breathable plate seals and incubated at 37°C for 48 hours. Following the incubation period, bacteria were streaked onto agar plates with four delineated quadrants. Each bacterial strain included a positive control (CHX), a negative control (broth), and one of two of the dilution series. Each condition was performed 3 times using different colonies.

### 2.3. Cell Culture and SDF Application

HGF-1 cells (CRL-2014, ATCC, Manassas, VA) were cultured in 75 cm^2^ tissue culture flasks in growth media using Dulbecco's Modified Eagle's Medium (DMEM) supplemented with 10% fetal bovine serum (FBS) and 5% penicillin-streptomycin (P/S). Cells were incubated at 37°C in microaerophilic conditions with change of media every 3 days. HGF-1 cells, from passage 2, were seeded in 6 well plates with a concentration of 3.1 × 10^4^ cells/cm^2^ in growth media. 1 *μ*L of 0.394% SDF was applied into the cell culture medium. Cells were incubated at room temperature and washed with PBS (1x) and exposed to 10 *μ*L of trypan blue to check for cell viability. A SPOT Idea 3.0 Mp Color Digital Camera, recorded a 60-second time lapse from the time of application of SDF; no changes were noted beyond 30 seconds. SDF application to HGF cells was performed with six independent replicates.

### 2.4. Human Skin Equivalent Culture

Three-dimension models of human skin equivalent (HSE) consisting of a basal acellular collagen layer, a collagen matrix with human dermal fibroblasts, and human epidermal keratinocytes (human neonatal foreskin keratinocytes) were obtained from Dr. Johnathan Garlick (Tufts School of Dental Medicine, Boston, MA) [16]. HSE constructs were engineered to have a liquid air interface, allowing for full differentiation of epithelial layers with multiple cell types. HSE used in these studies was matured for 10 days and exhibited basement membrane, normal basal, spinous, granular, and keratinized layers [17]. Cornification medium was changed every two to three days. Three drops of 3 *μ*L of 38% SDF were applied directly onto the 3D tissues and harvested at 12 hours, 1, 3, 5, and 10 days posttreatment. Plates were photographed after applying SDF and observed for ten days.

At the end of treatment, samples were dissected away from the inserts using fresh 12 blade scalpels and tissue forceps. Tissue was divided into three equal parts and placed in tissue processing cassettes (Fisher Scientific Electron Microscopy Sciences CellSafe Biopsy Insert). Samples were fixed overnight in 4% formalin made in phosphate buffer saline (PBS). Samples were then washed 3 times with PBS and dehydrated through increasing gradients of alcohol, using standard sequential dehydration techniques. Tissues were then embedded, with tissue standing on edge, in hot paraffin wax. 6 *μ*m sections were mounted onto positively charged slides (Thermo Scientific™ Shandon™ ColorFrost™ Plus Slides).

### 2.5. Histology H&E and TUNEL Stain

Six slides of each H&E and TUNEL staining were processed for each time point observed. Slides were incubated for 15 minutes at 60°C and processed for H&E staining per standard protocol. After rehydration, slides were placed in Mayer's hematoxylin solution for two minutes. Slides were rinsed with distilled water and acid alcohol prior to placement into 1% lithium carbonate, where slides turned into purple-bluish hue. Slides were quickly dipped in 80% ethanol prior to exposure to eosin for 3 minutes. Finally, slides were dehydrated back through increasing concentrations of ethanol to xylene. Slides were mounted with VectaMount mounting media. Additional slides were processed for apoptosis staining following the manufacturer protocol (Thermofisher Scientific) using Click-iT™ TUNEL Colorimetric IHC Detection Kit. Histological images were taken in brightfield using a Nikon Eclipse E600 Fluorescence Microscope and SPOT Idea 3.0 Mp Color Digital Camera.

## 3. Results

**Figure 1:**
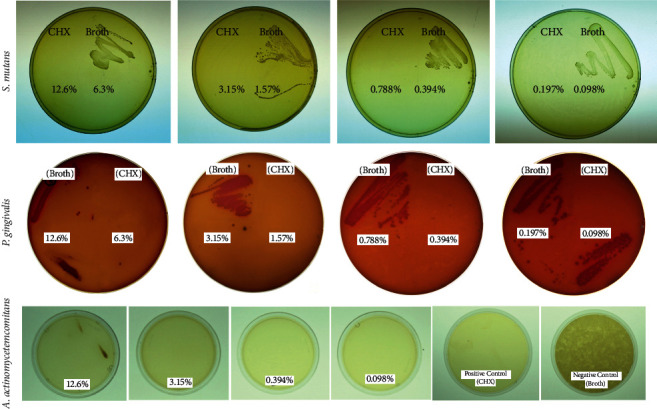
Effect of SDF on bacterial growth. First row, *S. mutans* was streaked on brain heart infusion agar plates. SDF dilutions are annotated in bottom two quadrants of agar plates. SDF inhibited *S. mutans* growth at all concentrations tested. Second row, *P. gingivalis* was streaked on tryptic soy with 5% defibrinated sheep blood agar plates. Note the SDF precipitate artifact in 12.6% concentration lower left quadrant. Colonies growth was noted in quadrants with 0.197% and 0.098% SDF. Third row, *A. actinomycetemcomitans* was streaked onto brain heart infusion agar plates. Due to the need for longer incubation periods and the fast growth and spread of *A. actinomycetemcomitans* on agar plates, colonies could not be cultured by quadrant as with the other bacteria. Colonies were instead inoculated on separate Petri dishes. SDF inhibited *A. actinomycetemcomitans* growth at all concentrations tested. All concentrations were performed in triplicate, and the process was repeated twice.

In the first series of experiments, we tested the effect of SDF on the growth of periodontal bacteria (Figure 1). Bacterial viability was assessed by inoculation of each culture condition on agar plates. Positive and negative controls of chlorhexidine and broth reflected expected outcomes of growth and inhibition for each bacterial colony, ensuring sound protocol. As expected, *S. mutans* growth was completely inhibited by SDF, showing bacterial inhibitory effects of SDF at all concentrations. *A. actinomycetemcomitans* growth was completely inhibited even with lowest SDF concentration (0.098%). On the other hand, *P. gingivalis* growth was inhibited at higher concentrations of SDF, but not by the lower concentrations (0.197% and 0.098%).

**Figure 2:**
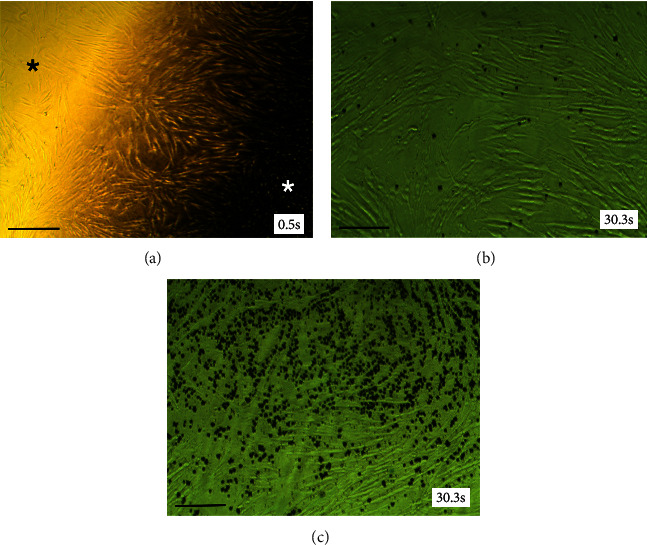
Effect of SDF on 2D cultures of human gingival fibroblasts (HGF). (a) SDF front showing HGF-1 cells with zone of affected cells (white asterisk) upon application of 1 *μ*L of 0.394% SDF and peripheral untreated cells (black asterisk). (b) Sparsely infiltrated region of dead cells in peripheral untreated region. Time lapsed indicated in lower right corner. (c) Dense areas of dead cells (appearing as blue, round cells) of treated region. (Scale bar = 100 *μ*m.).

**Figure 3:**
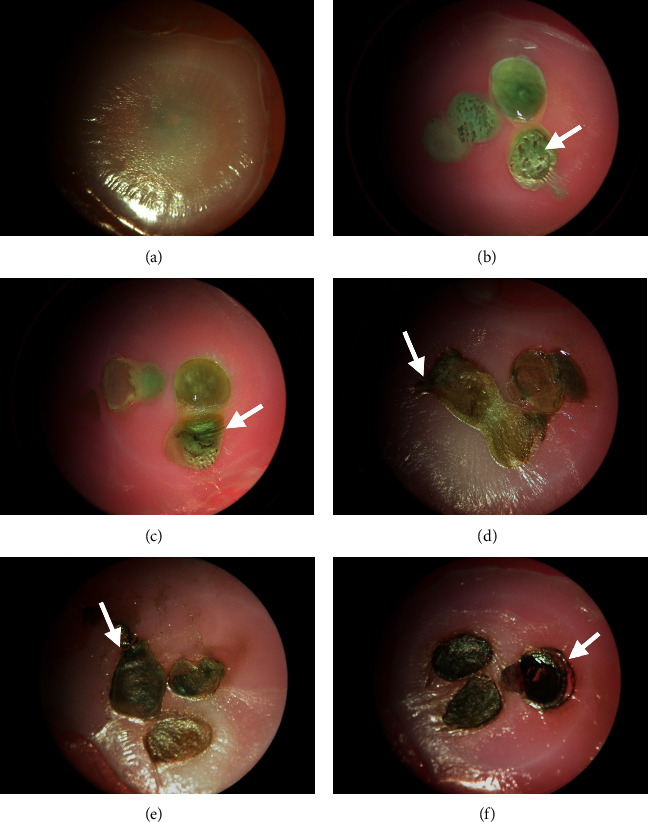
Effect of SDF on HSE constructs. The HSE constructs used are grown on Fisher CellSafe Biopsy Inserts, which are 18 × 18 mm in dimension. (a) Control untreated construct. B through F are treated constructs that received 3 *μ*L of 38% SDF and incubated for: (b) 12 hours: center of lesion is white-gray and reticulated with smooth surrounding tissues; (c) 24 hours: color of lesion shifts to a more grayish hue with slight contraction noted across surface of lesion and surrounding tissues; (d) 3 days: dark pigmentation starts to develop with an induration of the affected keratinized layer of epithelium; (e) 5 days: Continued darkening of pigmentation with inflammation in surrounding epithelium; and (f) 10 days: Complete induration noted with blackened crust and contraction of eschar from underlying tissue (white arrow).

We next tested the effect of SDF on fibroblasts viability grown as 2D monolayers (Figure 2) or as 3D HSE (Figure 3). Addition of 1 *μ*L SDF (0.394%) caused a rapid change in cell morphology of HGF-1 monolayers indicating cell death. HGF-1 cells prior to inoculation are depicted by the dark asterisk (Figure 2(a)), whereas affected region (white asterisk) is shown on the other side of the SDF front. Following the application of trypan blue, peripheral cells untouched by the SDF exposure show sparsely infiltrated dead cells (Figure 2(b)), whereas Figure 2(c) shows a dense infiltrate of dead cells (blue, round cells) in affected region.

**Figure 4:**
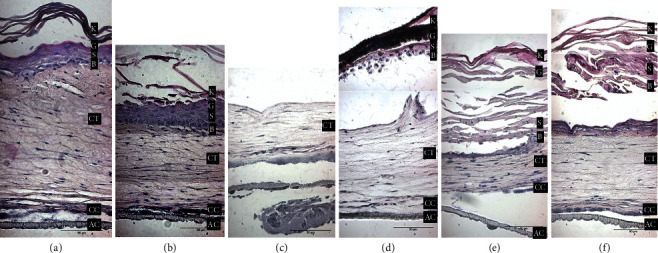
Histological examination of H&E stained HSE constructs. The following annotation abbreviations are used: acellular collagen (AC), cellular collagen (CC), connective tissue (CT), basal layer (B), spinosum (S), granular (G), keratin (K). (a): Untreated construct. (b) 12 hours: intact epithelium noted, with the exception of the superficial layers of the stratum corneum. (c) 24 hours: Epithelial layer of the 24-hour sample was lost during processing leaving only the connective tissue and underlying collage portion. (d) 3 days: Epithelium has lifted with contracted stratum corneum with largely unaffected connective tissue. (e) 5 days: Stratum granulosum is very spread out with intact stratum basale. Connective tissue also looks unaffected. (f) 10 days: Epithelium has also lifted away from connective tissue and appears very deformed and nearly indistinguishable between various epithelial layers with change in morphology. Connective tissue appears unaffected. (Scale bar = 50 *μ*m).

**Figure 5:**
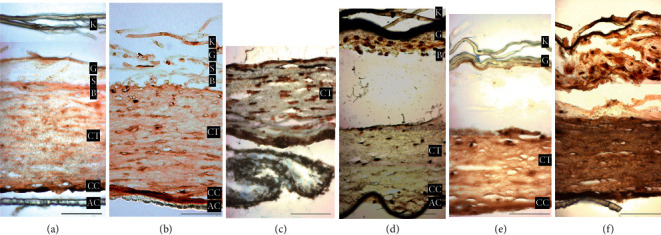
TUNEL staining for apoptotic cells. The following annotation abbreviations are used: acellular collagen (AC), cellular collagen (CC), connective tissue (CT), basal layer (B), spinosum (S), granular (G), keratin (K). Apoptosis of keratinocytes and fibroblasts in HSE was assessed with TUNEL staining—apoptotic cells nuclei will stain with dark (a) Control untreated construct shows minimal apoptosis occurring among fibroblasts in the most superficial region of the connective tissue; no epithelial cells stained for apoptosis. (b) 12 hours: Apoptotic cells noted throughout all nucleated layers of the epithelium and within the underlying connective tissue. (c) 24 hours: Missing epithelial layer lost during processing, but fibroblasts staining for apoptosis are limited to first few layers of connective tissue. (d) 3 days: Basal and suprabasal layers of epithelium are adherent to keratinized layer above and all stain dark for apoptosis along with superficial layer of connective tissue. (e) 5 days: Fibroblasts stain for apoptosis throughout all regions of connective tissue (f) 10 days: Fewer apoptotic cells that are limited to the first few cell layers of connective tissues. (Scale bar = 50 *μ*m).

HSE were either left untreated or received drops of a 38% SDF solution. Figure 3 compares visual appearance of untreated HSE (control) with constructs exposed to three drops of 38% SDF, followed for 12 hours, 1, 3, 5, and 10 days. After 12 hours, lesion surfaces appeared reticulated, and surrounding tissues are smooth (Figure 3(b)). By 24 hours, more retraction of surface lesion surrounding tissues were evident (Figure 3(c)). After 3 days, dark pigmentation developed with an induration of the affected keratinized layer of epithelium showing an eschar (Figure 3(d)). Lesions now developed a defined boundary with erythematous margins, showing inflammation extending into contacted epithelium. No significant changes were observed by day 5 aside from continued darkening of pigmentation and appearance of inflammation in neighboring epithelial regions (Figure 3(e)). After 10 days, the lesion formed a blackened crust that peeled away from borders due to the extent of contraction within the lesion (Figure 3(f)). It should be noted that, for all these experiments (Figures 3–5), there was only one control group (at one given time point) which is a study limitation.

HSE were fixed, embedded in paraffin, and sections were stained with H&E. As shown in Figure 4(a), in the ascending order, the different layers of HSE constructs: acellular collagen (AC), cellular collagen (CC), connective tissue (CT), and differentiated layers of the epithelium. All samples, control included, have some commonalities: fibroblasts (deep in the connective tissue) exhibit a flat morphology, indicating viability; fibroblasts (towards the surface) exhibit a rounder appearance, suggesting an apoptotic state. The epithelial layer of the 24-hour sample (Figure 4(c)) was lost during processing, leaving connective tissue and collagen. The 12-hour sample exhibits an intact epithelium, with the exception of the superficial layers of the stratum corneum. However, from days 3–10, the epithelial layer is completely lifted, adhering to the contracting stratum corneum.

Apoptosis of keratinocytes and fibroblasts in HSE following exposure to SDF was assessed with TUNEL staining. Nontreated HSE was stained to provide a negative control (Figure 5(a)) showing minimal apoptosis occurring among fibroblasts in the most superficial region of the connective tissue; no epithelial cells were stained for apoptosis. Tissues exposed to SDF (Figure 5(b)–5(f)) showed apoptotic cells throughout all nucleated layers of the epithelium and underlying connective tissue. Treated tissues harvested earlier show apoptotic cells extending throughout the upper half of the connective tissue portions (Figure 5(b)–5(d)). Samples taken at later time points (day 5 and day 10) show fewer apoptotic cells that are limited to the first few cell layers of connective tissues.

## 4. Discussion

The use of SDF as a caries arresting agent is valued for its bactericidal effects on cariogenic bacteria, ability to harden softened dentin, and alleviation of dentinal hypersensitivity [13] each relating back to SDF's synergistic effects of silver and fluoride ions [18]. The effect of SDF may also hold promising implications within periodontics. Like cardiology, periodontal disease involves a transition to bacterial dysbiosis [11], collagen breakdown [19, 20], and sensitivity—related to breakdown in dentin nearing the pulp chamber or exposure of dentin with clinical attachment loss [21, 22].

In vitro studies have shown bactericidal effects of SDF on Gram-positive, cariogenic bacteria such as *S. mutans, S. sobrinus, L. acidophilus, L. rhamnosus*, and *A. naeslundii* [18]. The bactericidal efficacy of the drug is attributed to the silver ion's cationic effect in disturbing negatively charged bacterial components of Gram-positive bacteria. Our findings are in agreement with the published report showing that *S. mutans* growth was completely inhibited by SDF, even with the lowest dilution (0.098%) used.

While no current evidence exists regarding the effect of SDF on Gram-negative bacterial structures, a hypothesis can be drawn regarding silver's cationic properties due to its negatively charged outer membrane [23]. Studies using cationic agents (magnesium or chlorhexidine) have shown competitive ionic binding onto negatively charged lipopolysaccharides, affecting ion-pairing, resulting in an electrostatic charge, affecting the lateral packing and permeability of the outer membrane [16, 24]. Advances have been made in the field of silver nanoparticles (AgNP). AgNP are <10 nm in diameter and are essentially micelle aggregate structures of silver ions that may readily attach to bacterial cell walls of both Gram-positive and Gram-negative bacteria, causing membrane permeability [17].

The antimicrobial effects of silver on Gram-negative periodontal pathogens have been demonstrated with silver nitrate on *P. gingivalis* and *A. actinomycetemcomitans* [25]. It was postulated that silver may interfere with sulfhydryl bonds of bacterial enzymes [26]. While the specific nature remains unknown, the bactericidal effects of SDF on Gram-negative bacteria has been demonstrated in this pilot study. High concentrations of SDF (12.6% to 0.394%) resulted in complete inhibition of *P. gingivalis* growth, but lower concentrations (0.197% and 0.098%) were ineffective. Previous findings using silver nitrate report a 3log_10_ reduction in *P. gingivalis* at a concentration of 0.5 *μ*g/mL^25^—a much lower concentration than the most diluted concentration of SDF used in our study (1.97 *μ*g/mL), suggesting higher potency of silver nitrate. On the other hand, growth of *A. actinomycetemcomitans* was completely inhibited even at lower concentrations of SDF.

In an *in vitro* study with cultured fibroblasts, silver has been shown to be cytotoxic in silver nitrate at 14 × 10^−5^% with a contact time of 2 hours [27]. Likewise, SDF incubated in hydroxyapatite discs was shown to be cytotoxic to human gingival fibroblasts at 0.01%. It retained its cytotoxic effects after 9 weeks of rinsing with artificial saliva [28]. SDF cytotoxicity may be related to increase in oxidative stress or lipid peroxidation [29]. Our results concur with previous 2D *in vitro* studies of human gingival fibroblasts: toxicity is instantaneous and severe. Upon application of 1 *μ*L of 0.394% SDF, human gingival fibroblasts showed immediate disruption of the attachment process. Fibroblasts transformed from a well-attached, smooth cell with thin cytoplasmic extensions to rough cells with broad cytoplasmic extensions unable to exclude trypan blue, indicating cell death. However, 2D matrices poorly represent the anatomy found intraorally in which fibroblasts would be safely embedded within a connective tissue structure. We therefore used a 3D HSE construct to test the effect of SDF. All treated regions of the epithelium formed a scab-like layer with apoptosis noted in the suprabasal and basal layers of the epithelium, along within the superficial 50 *μ*m of the connective tissue layer but not in the deepest layers of connective tissue. Apoptotic cells were noted deep into the middle third of the connective tissue (12 hours and 24 hours) and were noted even deeper within the bottom third of the connective tissue (day 3). However, a shift was noted between days 5 and 10, where the apoptotic cells are only found in the most superficial layers of the connective tissue. This shift in apoptotic cells may indicate a shedding or regenerative capacity of the HSE where connective tissue layers continue to develop at the deepest layers and shed the superficial layers, resulting in an upward trend of apoptotic cells.

Human neonatal foreskin fibroblasts and keratinocytes constructs used herein adopt morphology very similar to that of the fully differentiated gingiva. Although this HSE model differs from human oral epithelium in that the epithelial layer does not form invaginations into the underlying lamina, but lies in a flat, linear topography, this model is still suitable to study the effect of SDF on fibroblasts and epithelial cells. In an effort to better imitate intraoral environments, future studies may include an artificial saliva media to better imitate an intraoral environment. Another study limitation is that the experiments on the HSE model were performed only once. This study sought to identify the effects of SDF on periodontal pathogens, and the qualitative nature of the design held limitations. Initial study designs involved quantification of bacterial viability with exposure to SDF (via optical density), but this form of quantification was muddied by the precipitate of Ag^+^ ions in the culture medium. To note, SDF is a highly reactive material to any medium it is in direct contact with, with minimal penetrative value. In the case of 2-D HGF-1 cell culture, SDF spread so rapidly that all cells in contact to the solution were dead within 30 seconds. The choice for 0.394% of SDF in this portion of the study stemmed from the results that indicated limited effects of 0.197% SDF on *P. gingivalis*. No quantitative evaluation was necessitated due to the complete cell cytotoxicity observed upon application of the solution.

In summary, our study demonstrates bactericidal effect of SDF on Gram-negative bacteria. There are promising implications for the medicament in actively progressing periodontal lesions. This study also shows the limited extent of damage caused by SDF within the epithelium and surface portion of connective tissue in a 3-D HSE construct, which may suggest a limited range of 50 *μ*m of destruction within connective tissue.

## Data Availability

Data will be available from the corresponding author upon request.
